# Robotic Omental Flap Harvest for Complex Thoracic Defects: Case Series and Review of the Literature

**DOI:** 10.3390/medsci13040264

**Published:** 2025-11-12

**Authors:** Susana Fortich, Camila Franco-Mesa, Jennifer Den, Gabriel De La Cruz Ku, Gal Levy, Roman Petrov

**Affiliations:** 1 Department of General Surgery, University of Texas Medical Branch, Galveston, TX 77555, USA; sufortic@utmb.edu (S.F.); camfranc@utmb.edu (C.F.-M.); jeden@utmb.edu (J.D.); 2 Department of General Surgery, University of Massachusetts, Worcester, MA 01605, USA; gdelacruz@cientifica.edu.pe; 3 Division of Cardiothoracic Surgery, Department of Surgery, Howard University, Washington, DC 20060, USA; gal.levy@howard.edu; 4 Division of Cardiothoracic Surgery, Department of Surgery, University of Texas Medical Branch, Galveston, TX 77555, USA

**Keywords:** robotic omental flap, chest wall reconstruction, robotic surgery

## Abstract

Objective: The omentum is a highly vascularized and immunologically active tissue with significant regenerative potential. Despite its versatility, its use has traditionally been limited to intra-abdominal applications due to access challenges. Conventional open harvest requires laparotomy, and laparoscopic techniques are hindered by limited visualization and poor ergonomics. We describe the use of robotic-assisted omental flap harvest for thoracic reconstruction, offering a minimally invasive alternative. Methods: A retrospective review was conducted of patients who underwent robotic omental flap harvest for intrathoracic reconstruction at a single-center institution between January 2023 and January 2024. Data collected included demographics, indications, surgical technique, operative details, and postoperative outcomes, with a focus on flap viability and complications. Additionally, a systematic review was conducted to evaluate current evidence and experiences with this type of technique. Results: Three patients underwent robotic omental flap harvest for indications including chest wall reconstruction and pleural space obliteration in infected thoracic cavities. The average robotic flap harvest time was 79 ± 13 min, with an estimated ± blood loss of 20 cc. The mean postoperative hospital stay was 10 days, influenced by the primary procedure and patient comorbidities. At an average follow-up of 8 months, all flaps remained viable, with no flap-related complications or losses. The systematic review demonstrated limited data in the current literature regarding this type of surgical approach. Conclusions: Robotic-assisted omental flap harvest is a safe, feasible, and effective technique for complex thoracic reconstructions. It provides a minimally invasive alternative to traditional harvest methods, with reduced morbidity and excellent clinical outcomes. This technique expands the reconstructive options for intrathoracic defects and infections.

## 1. Introduction

The greater omentum has long fascinated surgeons not only for its anatomic presence but also for its unique biological properties. Early descriptions of its use in surgery date back more than a century, with pioneering surgeons recognizing its ability to control infection and promote wound healing in otherwise hostile environments. Since then, the role of the omentum has broadened considerably, extending into reconstructions of the head and neck, breast, extremities, and abdomen. Its consistent vascularity and immunomodulatory capabilities provide advantages unmatched by many other flaps, particularly in settings where local tissue is either inadequate or compromised. The omentum is a versatile organ, composed of a dual layer of mesothelium with adipose tissue, fibroblasts, pericytes, and leukocytes [1]. This 300 to 1500 cm^2^ structure originates over the greater curvature of the stomach and descends to the pelvis, draping over intraperitoneal organs [1,2]. Although underappreciated, the omentum functions as an independent organ with specific functions in tissue regeneration and immunologic regulation [1]. The main intrinsic structures are glomeruli-like conformations termed “milky spots” [1,2]. These units possess an abundant vascular supply and a tailored phagocyte system that rests in a fenestrated scaffold [2]. Nutrients, lymphocytes, and macrophages migrate through these milky spots, contributing to wound healing and providing anti-inflammatory and antibacterial functions [2]. Given its rich vasculature, abundant lymphatic system, and immunomodulatory features, this organ is a reliable source for tissue reconstruction, both as a pedicle or a free flap [2]. Nowadays, applications for omental flap use range from wound coverage to lymphedema treatment [3].

The omentum’s vascular supply primarily stems from the right and left gastroepiploic arteries, which travel along the greater curvature of the stomach. These arteries form an anastomotic arcade that supports the viability of the omentum when mobilized as a flap. During flap harvest, the omentum is typically based on the right gastroepiploic artery for transposition into the right hemithorax or substernal space, whereas the left gastroepiploic artery may be preserved or divided depending on flap orientation. In left-sided reconstruction, the flap can be based on the left gastroepiploic artery. Venous drainage mirrors the arterial pattern, with corresponding gastroepiploic veins draining into the superior mesenteric and splenic veins. Adequate mobilization requires careful preservation of the vascular pedicle and meticulous dissection from the transverse colon and greater curvature to avoid devascularization. The flap’s length and versatility allow for extensive reach, making it suitable for covering thoracic defects through a transdiaphragmatic or substernal tunnel [4].

The omentum’s central location allows for tissue reconstruction in the head, neck, thorax, abdomen, and extremities [2]. It is especially recognized as a salvage option for deep sternal wound infections or thoracic defects when local reconstruction options are insufficient or have failed [5]. While omental flaps have been used as a reliable option for the past century, their intra-abdominal nature is one of the greatest limiting factors. Traditional flap harvest mandates a laparotomy incision, carrying its own risks and morbidity, such as delayed recovery, intrabdominal organ injury, adhesions, incisional hernia, wound dehiscence, infection, and others [6].

Over the past few decades, minimally invasive approaches, such as laparoscopy and, more recently, robotic technology, have been increasingly used in clinical practice. While laparoscopic omental flap harvest has been described, factors such as limited spatial visualization, restricted range of motion, patient-related anatomic constraints, and poor ergonomics limit its use. However, modern robotic technology, featuring augmented dexterity and safety features such as three-dimensional vision, fully articulated instruments, and improved ergonomics, facilitates flap harvesting without the technical limitations encountered during laparoscopy. Due to its advantages, robotic technology is associated with lower conversion rates to open surgery, reduced blood loss, and shorter length of stay for select procedures [7]. Although robotic omental flap harvest is a novel concept that has been rarely described in the literature, it is an invaluable option for reconstruction that addresses the limitations and risks of traditional omental flap harvest.

The present case series builds on this foundation by providing a detailed description of the robotic omental flap harvest technique and its successful application in three distinct thoracic reconstructions. By situating our findings within the broader context of reconstructive and robotic surgery, we aim to illustrate the clinical value of this approach and contribute to the growing body of evidence supporting its role in complex thoracic surgery.

## 2. Patients and Methods

### 2.1. Case Series

A retrospective review was conducted for all patients who underwent robotic omental flap harvest for thoracic reconstruction at our single-center institution from January 2023 to January 2024. Salvage procedures were included, as well as sternal and chest wall reconstructions. All patient information, including preoperative comorbidities, prior procedures, diagnoses, indications, operative details, and follow-up data, was collected from the medical records. The primary endpoint was to describe the surgical technique and postoperative outcomes. Patient follow-up was conducted until May 2025. Given the nature of the study and the small sample size, no statistical significance tests were performed. This retrospective study used anonymized data from hospital records. According to the institutional policy, ethical approval and informed consent were not required for this type of research.

Inclusion criteria were patients requiring intrathoracic reconstruction in whom local flaps (e.g., pectoralis, rectus abdominis, or latissimus) were considered inadequate or had failed. Exclusion criteria included patients with severe intra-abdominal adhesions prohibiting safe laparoscopic or robotic access, hemodynamic instability precluding pneumoperitoneum, or previous gastric surgery compromising gastroepiploic vessel supply. Patients were evaluated preoperatively with cross-sectional imaging to assess defect size and the anticipated reach of the omental flap, and the surgical approach was selected in a multidisciplinary conference.

### 2.2. Perioperative Management

All patients received standard perioperative care, including prophylactic broad-spectrum intravenous antibiotics prior to skin incision, with adjustments based on culture results when infection was present. Deep venous thrombosis (DVT) prophylaxis with sequential compression devices and subcutaneous heparin was initiated unless contraindicated. Nutritional optimization was emphasized, and in cases of preexisting malnutrition, supplemental enteral or parenteral nutrition was initiated prior to reconstruction.

Postoperative pain management followed an Enhanced Recovery After Surgery (ERAS) protocol, consisting of multimodal analgesia with acetaminophen, nonsteroidal anti-inflammatory drugs (NSAIDs), and regional anesthesia when feasible. Early mobilization and pulmonary hygiene were emphasized, particularly for patients undergoing intrathoracic procedures, to minimize the risk of atelectasis and pneumonia.

### 2.3. Operative Procedure

#### 2.3.1. Positioning, Landmarks, and Port Placement

All procedures were performed using the Da Vinci Xi surgical platform (Intuitive Surgical, Sunnyvale, CA, USA). Patients were positioned supine with both arms tucked alongside the body. The chest and abdomen were prepped and draped in the usual sterile fashion. Four 8 mm robotic ports were placed in a horizontal line, two inches above the umbilicus. The camera port (arm 2) was placed 2 cm to the left of the midline. Arm 1 was positioned in the right mesogastrium at the midclavicular line, arm 3 at the left midclavicular line, and arm 4 at the left anterior axillary line. A minimum distance of 8 cm was maintained between ports to prevent collisions of the robotic arms. A 30-degree camera was used in arm 2, a fenestrated bipolar grasper in arm 1, Vessel Sealer Extend or SynchroSeal in arm 3, and a tip-up grasper in arm 4. The patient was positioned in a 20-degree reverse Trendelenburg position with 15 mmHg of pneumoperitoneum induced, and the Da Vinci robot was docked.

#### 2.3.2. Flap Harvest

Upon entering the abdomen, a general inspection was conducted to identify gross anatomy and any atypical or unexpected findings. The quality of the omentum was examined, emphasizing its size, laxity, and the presence of adhesions. Previous abdominal surgeries do not preclude the use of the omentum; however, in the presence of postoperative adhesions, extensive lysis might be required for mobilization. Subsequently, the omentum is elevated cranially and mobilized off the transverse colon attachments over the avascular plane. Planned reconstruction of the right chest or substernal area requires constructing the omental flap based on the right gastroepiploic artery. Left chest flaps can be based on the left gastroepiploic artery. Dissection for flap preparation starts distally, mobilizing the omentum off the greater gastric curvature while preserving the integrity of the gastroepiploic artery. Flap transposition can be accomplished through the substernal area by dividing the sternal portion of the diaphragm or directly into the pleural cavity by mobilizing the diaphragm away from its costal attachments.

Based on the required size for translocation, mobilization from the spleen and along the greater curvature of the stomach was performed by extending the avascular plane while ensuring that the pedicle and surrounding vasculature were not injured. Once the dissection was complete, with sufficient omental tissue for intrathoracic transposition, the flap was secured to the diaphragm with a Vicryl suture to prevent displacement.

### 2.4. Tunneling

In cases of substernal transposition, the falciform ligament was mobilized from the abdominal wall as needed to create a tunnel for flap delivery. The xiphoid was identified, and the sternal portion of the diaphragm posterior to the xiphoid was divided. A passage communicating into the anterior mediastinum was developed. For the sternal reconstruction case, the flap was brought to the open sternal wound through a small subxiphoid incision made in the previous sternal incision. For the two cases that involved obliteration of the right pleural cavity, access was obtained through the mediastinum, and the flap was delivered to the right pleural cavity.

### 2.5. Inset

In the case of sternotomy dehiscence, the omental flap was draped over the defect and secured with deep dermal sutures. Subsequently, a 1/12,000 inch split-thickness skin graft was placed over the flap and sealed with Artiss Fibrin Sealant. In instances of pleural space obliteration, the flap was secured to the edge of the diaphragm defect using Vicryl sutures.

### 2.6. Literature Review

This review was conducted in accordance with the Preferred Reporting Items for Systematic Reviews and Meta-Analyses (PRISMA) 2020 guidelines. No review protocol was registered in PROSPERO or any other database. The databases PubMed (MEDLINE), Scopus, and the Cochrane Central Register of Controlled Trials were searched from inception through February 2024. The complete search strategy for PubMed was as follows: (“robotic” OR “robot-assisted”) AND (“omental flap” OR “omental harvest” OR “omentoplasty”) AND (“reconstruction” OR “minimally invasive” OR “thoracic” OR “flap harvest”).

Equivalent syntax was adapted for Scopus and Cochrane Central. Filters were applied to restrict results to English and Spanish publications and human subjects.

Study selection was performed independently by two reviewers (SF and CF) who screened all titles and abstracts. Full texts of potentially eligible studies were then reviewed for inclusion. Disagreements were resolved by consensus with a senior reviewer (RP).

Data extraction was carried out independently by two reviewers using a standardized electronic form, collecting patient demographics, surgical indications, operative details, and outcomes.

Given that all included publications were case reports or small case series, a formal risk-of-bias assessment was not applicable. Data were synthesized qualitatively, summarizing patient characteristics and outcomes without meta-analysis due to heterogeneity and limited sample size.

**Figure 1:**
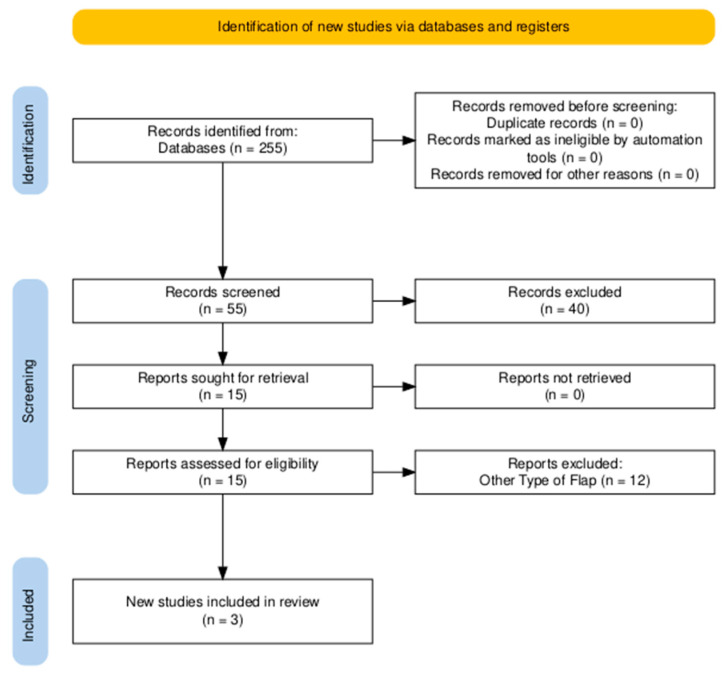
PRISMA diagram of identification of studies via databases [8].

The review process and study inclusion are summarized in Figure 1 (PRISMA 2020 flow diagram) [8].

No review protocol was registered in PROSPERO or any other registry. The review followed the Preferred Reporting Items for Systematic Reviews and Meta-Analyses (PRISMA) 2020 guidelines to ensure methodological transparency and reproducibility (Appendix A).

Given the qualitative nature of the synthesis and the inclusion of only case reports and small case series, formal assessments of reporting bias and certainty of evidence were not performed. However, potential publication bias was mitigated by conducting a comprehensive search across multiple databases without date restrictions.

## 3. Results

### 3.1. Institutional Experience

Three cases of omental flap reconstruction with robotic harvest were performed during the study period. The average proceduroperative operative duration was 265 ± 46 min, with an estimated blood loss (EBL) of 335 cc, including 79 ± 13 min for robotic flap harvest. The average length of hospital stay was 10 days, largely influenced by underlying comorbidities rather than flap-related morbidity. The average follow-up period after surgery was 8 months. At follow-up, no patient experienced flap loss or required reoperation, and all three surgical defects were successfully closed without recurrence.

One superficial wound complication was observed in Patient C, characterized by a 2 cm × 2 cm superficial dehiscence of the skin graft, managed conservatively with wound vacuum-assisted closure. No intra-abdominal complications, such as bleeding, visceral injury, or incisional hernia, were observed. Importantly, there were no conversions from robotic to open harvest, underscoring the feasibility of the minimally invasive approach in our early institutional experience. A summary of patient characteristics and demographics can be seen in Table 1.

**Figure 2:**
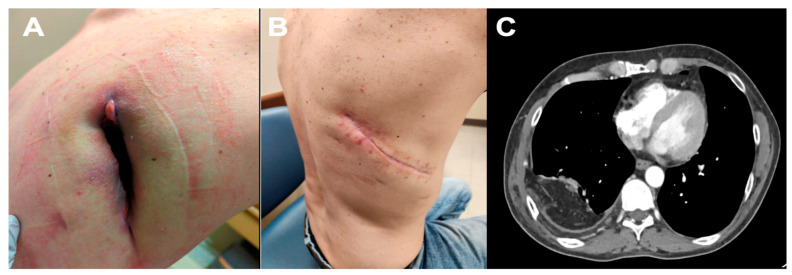
Patient A. (**A**)—15 × 4 cm chest wall defect after previous thoracostomy for the entrapped lung with bronchopleural fistula. (**B**)—Complete closure of chest wall defect. (**C**)—CT thorax with IV contrast demonstrating location of omental flap with coverage of chest wall defect.

**Patient A**: A 45-year-old male with a medical history (PMH) of alcohol abuse, a former smoker with a history of 27 pack-years, depression, type one diabetes mellitus, and bronchopleural fistula. The patient previously underwent open thoracotomy, empyema drainage, decortication, and Eloesser flap five months prior to his clinic presentation. He reported serosanguinous fluid drainage from the chest wall defect. On physical examination, the patient had an 15 × 4 cm posterior-lateral right chest wall defect with a persistent broncho-cutaneous fistula (Figure 2). Options for long-term wound care versus reconstructive surgery were presented, and the patient chose omental flap transposition. A computed tomography (CT) scan of the chest demonstrated a defect in the right posterior chest wall extending into the pleural space. Consequently, a robotic-assisted omental flap harvest, Eleossar flap takedown, reopening of the prior thoracotomy, partial resection of the 7th rib, diaphragmotomy, intrathoracic transposition of the omental flap, surgical closure of the bronchopleural fistula with omental flap buttressing, and complex chest wound closure with a myocutaneous advancement flap measuring 15 × 4 cm were performed, along with right chest tube placement and Jackson Pratt (JP) placement beneath the latissimus dorsi muscle at the top of the chest wall. The robotic omental flap harvest was conducted in a supine position with robotic ports placed as previously described. During the thoracic portion, the patient was transitioned into a left lateral decubitus position. The surgery lasted 285 min, with an EBL of 500 cc. The robotic omental flap harvest took 67 min. The postoperative course was uncomplicated, and the patient was discharged home on postoperative day (POD) 3. During the postoperative follow-up, the patient achieved an excellent result, with successful wound closure and outstanding functional outcomes.

**Figure 3:**
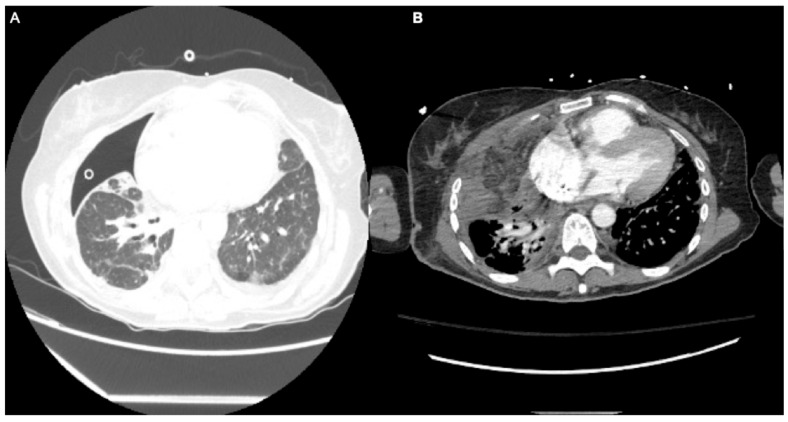
Patient B, (**A**)—CT chest with chronic interstitial lung disease, chronic parenchymal opacities, and chronic scarring throughout both lungs. (**B**)—Postoperative CT scan with omental flap in place.

**Patient B:** 56-year-old female with a medical history that includes psoriasis, idiopathic pulmonary fibrosis (on Humira/Stelara) (Figure 3), and recurrent right spontaneous pneumothorax with prolonged air leak. This condition required a robotic-assisted right apical wedge resection and decortication with pleurodesis. The procedure was complicated by an entrapped lung, persistent air leak, and empyema. A second video-assisted thoracoscopic surgery (VATS) was performed, which included complete lung decortication, debridement of the empyema, and partial pleurectomy. Despite these efforts, the patient’s air leak continued, failing all conservative interventions. The air leak persisted for months and did not respond to the placement of endobronchial valves. Surgical intervention was ultimately considered a last resort, and the patient opted for it. A robotic-assisted omental flap harvest with intrathoracic transposition, right thoracotomy, intrathoracic transposition of the serratus anterior flap, and modified thoracoplasty with partial resections of the right 2nd, 3rd, and 4th ribs were performed. The total duration of the procedure was 310 min with an EBL of 500 cc. The robotic omental harvest took 98 min. For the omental flap harvest, the patient was positioned supine and then transitioned to a left lateral decubitus position for the thoracotomy. Following the surgery, the air leak resolved. However, the postoperative course was complicated by respiratory failure, necessitating prolonged mechanical ventilation with reintubation after a failed extubation attempt. Eventually, the patient required a percutaneous tracheostomy on postoperative day 14 due to an inability to wean from mechanical ventilation, and a percutaneous endoscopic gastric (PEG) tube was placed for supplemental nutrition. The patient was discharged to a long-term acute care hospital (LTACH) with a referral for a lung transplant. She remains tracheostomy-dependent and requires CPAP at night.

**Figure 4:**
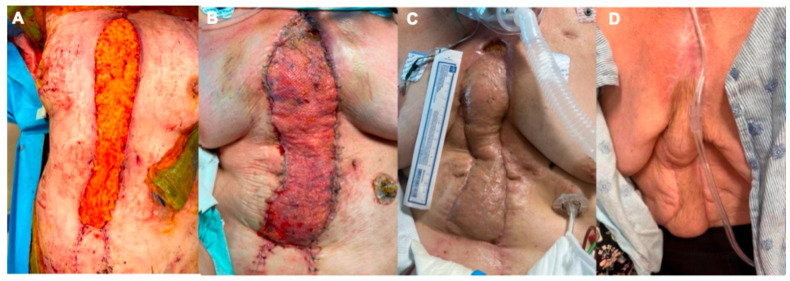
Patient C, sternal wound omental flap with STSG coverage. (**A**)—placement of the omental flap. (**B**)—Application of the STSG. (**C**)—POD 15 after wound flap closure. (**D**)—Eleven months after omental flap placement.

**Patient C:** A 64-year-old female with a past medical history of chronic obstructive pulmonary disease, tobacco abuse, and coronary artery disease underwent a coronary artery bypass graft. Her postoperative course was complicated by prolonged ventilation requiring tracheostomy, sternal dehiscence, mediastinitis, and osteomyelitis. Despite multiple debridements and a failed right vertical rectus abdominis myocutaneous flap (VRAM), the wound failed to heal. The decision was to proceed with an omental flap transposition for the sternotomy wound closure. The patient underwent robotic-assisted omental flap harvest based on the right gastroepiploic artery, mini-laparotomy, external transposition of the omental flap, bilateral pectoralis advancement flaps, and a split-thickness skin graft from the left thigh. The total duration of surgery was 201 min with an EBL of 70 cc. The robotic omental flap procedure duration was 74 min, with an EBL of 50 mL. The patient tolerated the procedure well, with 99% skin graft take (Figure 4). She was discharged to LTACH on POD13.

### 3.2. Systematic Review

A total of 255 studies were identified in the queried databases. From these, 15 full texts were reviewed, and 3 met the inclusion criteria for this study (Table 2). All of the studies were case reports [9,10] or case series [3] with a pooled number of 7 patients. No larger cohort studies or case–control studies were encountered in the literature. Of these seven procedures, six were free flaps, and one was a pedicle flap [3,9,10]. Most cases were performed for vascularized lymph node transfer for upper or lower extremity lymphedema (71%, *n* = 5). The remaining indications included chest wall reconstruction after radical chest wall mass resection (14%, *n* = 1) and defect closure after osteomyelitis (14%, *n* = 1) [3,9,10].

For vascularized lymph node transfer, four patients underwent a standard four-port harvest, and one patient underwent a single-port harvest, as reported by Frey et al. [3]. Lymph node clusters were identified with indocyanine green injected into the distal omentum [3]. All underwent omentum-free flap transfer to two sites (2 conjoined and three segmented flaps) [3]. Anastomoses were performed according to the location of the recipient site (thoracodorsal, radial, descending genicular, tibial, or inferior epigastric vessels) [3]. Complications included two episodes of cellulitis managed with oral antibiotics [3]. At an 8.8-month follow-up, the surgical sites had healed, and all patients showed improvement in lymphedema [3].

Regarding chest wall reconstruction, Day et al. [10] treated a 15 cm × 35 cm defect after angiosarcoma resection in a one-stage pedicle flap procedure. Four 7 mm robotic ports were placed transversely in line with the umbilicus, and the omentum was mobilized off the transverse colon. The pedicle was based on the gastroepiploic arch. The flap was covered with split-thickness skin graft, and a wound VAC was placed. During the postoperative period, the lateral side of the skin graft sloughed off, and this was managed with local wound care. The flap healed completely.

Lastly, one case of lower extremity reconstruction was described by Özkan et al. [9]. A 10 cm × 12 cm non-healing infected wound from osteomyelitis was covered with a free flap [9]. Three robotic and one laparoscopic assist port were used [9]. The pedicle was based on the right gastroepiploic vessels and anastomosed to the posterior tibial vessels [9]. A split-thickness skin graft was used for coverage. The postoperative course was uneventful, and the flap healed without complications [9].

## 4. Discussion

The omentum flap was first introduced for chest wall reconstruction after breast cancer excision in 1963 by Kiricuta et al. [11]. Applications of robotic omental harvest span from head and neck to thoracic reconstruction. Reports in plastic and reconstructive surgery have demonstrated feasibility in vascularized lymph node transfer for lymphedema and in salvage procedures for head and neck defects.

As previously mentioned, the omentum flap has long been recognized for its versatility in reconstructive surgery, particularly in managing complex thoracic and sternal defects. The use of omental flaps for chest wall reconstruction has been well-documented in both benign and malignant conditions, including after extensive oncologic resections, where their role in promoting wound healing and minimizing infection risk is especially valuable [12].

This case series presents three different thoracic indications for omental flap surgery. In the first case, the presence of a large defect communicating with the pleural cavity, coupled with a bronchopleural fistula, made the success of a myocutaneous flap unlikely. The omentum flap has the advantage of adapting to large wound defects, contains a rich vascular and lymphatic supply, and has angiogenic properties that may stimulate neovascularization in ischemic tissues. Additionally, due to its immunologic capacity and its anti-infection activity, the greater omentum was destined to play an integral role in this type of wound treatment [13]. Similar to our approach, Day et al. [10] reported on the closure of a large thoracic wall defect with a robotic-assisted pedicle omental flap. The described technique could have shortened the patient’s recovery time and improved pain control and cosmesis.

In the literature, cases of persistent air leak (PAL) with trapped lung and a bronchopulmonary fistula present a special clinical challenge. Common operative strategies for PAL include VATS or open thoracotomy with chemical [14] pleurodesis or pleurectomy. In cases of bronchopulmonary fistula, options range from direct stump closure with intercostal muscle reinforcement and/or acellular human dermal matrix (AlloDerm) placement to chronic open drainage or thoracoplasty with or without extrathoracic chest wall muscle transposition and, lastly, omental flap placement [15]. No reports of robotic omental harvesting have been published for the management of persistent air leak due to bronchopleural fistula.

Lastly, infected sternal wounds are known complications of cardiac surgery with an exceedingly high mortality rate. Numerous approaches have been described. Medical management includes culture-directed antibiotic therapy, while surgical approaches range from closed suction and irrigation to Vacuum-Assisted Closure (VAC), rigid sternal fixation, and flap coverage [16]. Omental flaps are typically reserved for situations where muscle flap coverage is insufficient or has failed [17], as seen in our experience. Although mortality rates in the literature range from 5% to 36%, these are based on variable follow-up and retrospective analyses, making comparisons challenging. Despite this, transposition of the omentum flap is still the treatment of choice for the closure of complex sternal wounds that are not amenable to muscle-flap coverage [17]. Management of extensive sternal and mediastinal infections with omental flaps offers a chance at salvage of these desperate situations. Robotic omental flap harvest offers a minimally invasive option for these procedures.

In the reported case series, robotic-assisted harvest of the omental flaps was successfully employed for sternal and intrathoracic infections, particularly when muscle flaps had failed and there was excessive dead space to be filled. Minimally invasive techniques facilitated the harvesting of the omental flaps, making this approach less morbid for patients and ideal for those with multiple comorbidities.

The ergonomic benefits of robotic technology also warrant consideration. Three-dimensional visualization, tremor filtration, and wristed instrumentation facilitate precise dissection and reduce surgeon fatigue, potentially shortening the learning curve compared with advanced laparoscopic techniques. Although initial capital and operative costs of robotic surgery remain high, these expenses may be offset by reductions in complications, hospital length of stay, and need for reoperations. Cost-effectiveness analyses specific to robotic omental flap harvest have not yet been performed but represent an important area for future investigation.

Moving forward, collaboration across institutions and the development of prospective registries will be critical to validate safety and long-term outcomes. Such efforts could also help define patient selection criteria, identify predictors of flap success, and clarify the relative benefits of robotic versus laparoscopic or open harvest. As robotic surgery continues to expand across specialties, robotic omental harvest is poised to become an increasingly important tool in the armamentarium of thoracic and reconstructive surgeons.

The main limitation of the available literature is the paucity of high-quality evidence. All identified studies consisted of case reports or very small case series, with heterogeneous indications and short follow-up times. No comparative or randomized studies have been published to date, making it difficult to draw firm conclusions regarding outcomes or establish standardized protocols. Despite these limitations, outcomes have been consistently favorable, with high flap viability and minimal complications reported across diverse indications

## 5. Conclusions

The versatility of the omental flap, combined with the precision of robotic surgery, allowed for effective defect closure and infection control in patients who were not candidates for traditional muscle flap procedures. These cases demonstrate the feasibility of a minimally invasive approach, reducing postoperative morbidity while achieving excellent long-term outcomes.

Beyond thoracic reconstruction, robotic omental harvest holds promise for a wide range of reconstructive applications, including vascularized lymph node transfer, complex mediastinal salvage, and defect coverage in irradiated fields. As robotic technology becomes more accessible and experience grows, its integration into reconstructive practice may redefine the standard of care for patients requiring omental flaps. Future investigations should focus on multicenter collaboration, prospective data collection, and cost-effectiveness analyses to validate the broader role of this technique in modern surgery.

## Figures and Tables

**Table 1:** Demographics, comorbidities, and outcomes of patients undergoing robotic omental flap harvest.

Patient	Age	Sex	Comorbidities	Indication	Prior Interventions	Procedure	Harvest Time (min)	EBL	LOS	Complications	Outcome
**A**	45	M	Type 1 DM, alcohol abuse, smoking history, depression	Bronchopleural fistula, chest wall defect	Thoracotomy, empyema drainage, decortication, Eloesser flap	Robotic omental flap harvest, thoracotomy, flap transposition, chest wall closure	67	500	3.0	None	Excellent wound healing, no recurrence
**B**	56	F	Idiopathic pulmonary fibrosis, psoriasis, immunosuppression	Persistent air leak, trapped lung, empyema	Robotic wedge resection, VATS decortication, endobronchial valves	Robotic omental harvest, thoracotom, serratus flap, thoracoplasty	98	500		Respiratory failure, tracheostomy, PEG	Air leak resolved, discharged to LTACH, trach-dependent
**C**	64	F	COPD, CAD, tobacco abuse	Sternal dehiscence, mediastinitis, osteomyelitis	CABG, debridements, failed VRAM flap	Robotic omental harvest, mini-laparotomy, pectoralis flaps, STSG	74	70	13.0	Superficial wound dehiscence (skin graft), managed conservatively	99% graft take, discharged to LTACH

Female (F), Male (M), diabetes mellitus (DM), chronic obstructive pulmonary disease (COPD), coronary artery disease (CAD), coronary artery bypass graft (CABG), vertical rectus abdominis myocutaneous flap (VRAM), video-assisted thoracoscopic surgery (VATS), percutaneous endoscopic gastrostomy (PEG), split-thickness skin graft (STSG), long-term acute care hospital (LTACH), estimated blood loss (EBL), length of stay (LOS).

**Table 2:** Systematic review robotic harvested omental flap.

Study	Age (y.o)	F/M	N of Patients	Pedicle/FF	Indication	Defect Size	LOS (d)	Outcomes	Complications
Day [10]	68	1/0	1	Pedicle	Chest wall defect after angiosarcoma resection	15 × 35 cm	-	Healing of the graft, cancer recurrence, hospice	Sloughing skin graft
Frey [3]	51.2	4/1	5	FF	Lymphedema	NA	5.2	Improvement of swelling	Cellulitis (2)
Özkan [9]	58	0/1	1	FF	Osteomyelitis LE	10 × 12 cm	12	Defect closure	None

F (female), M (male), FF (free flap), LOS (length of stay).

## Data Availability

The original contributions presented in this study are included in the article. Further inquiries can be directed to the corresponding author.

## Appendix A. PRISMA 2020 Checklist—“Robotic Omental Flap Harvest for Complex Thoracic Defects: Case Series and Review of the Literature”

**Item**	**Checklist Description**	**Location in Revised Manuscript**
1. Title	Identify the report as a systematic review.	Title page ‘…and Review of the Literature’
2. Abstract	Provide a structured summary following PRISMA 2020 for abstracts.	Structured abstract includes objective, methods, results, and conclusions.
3. Rationale	Describe rationale in the context of existing knowledge.	Introduction—first three paragraphs explaining background and clinical relevance.
4. Objectives	Provide explicit statement of objectives.	End of Introduction—‘aim to illustrate clinical value and contribute to evidence.’
5. Eligibility criteria	Specify inclusion and exclusion criteria and grouping.	Methods—Systematic Review section: case reports and small case series included; exclusions defined.
6. Information sources	List databases and last search date.	PubMed, Scopus, Cochrane Central—searched through February 2024.
7. Search strategy	Present full database strategies with filters and limits.	PubMed syntax and equivalent Scopus/Cochrane syntax included; filters for English/Spanish human studies.
8. Selection process	Describe screening methods and reviewers.	Two reviewers (SF, CF) screened independently; disagreements resolved by consensus (RP).
9. Data collection process	Specify how data were extracted and verified.	Two reviewers independently extracted using standardized form; discrepancies resolved by discussion.
10a. Data items—outcomes	List and define all outcomes sought.	Demographics, indications, operative details, outcomes, complications.
10b. Data items—other variables	List and define other variables.	Procedure type, approach, follow-up time; assumptions about missing data described.
11. Study risk of bias assessment	Describe bias assessment methods.	Formal assessment not applicable due to case report design; limitation acknowledged.
12. Effect measures	Specify outcome measures or summary statistics.	Qualitative synthesis; no quantitative effect measures.
13. Synthesis methods	Describe data preparation and synthesis approach.	Data qualitatively synthesized; no meta-analysis due to heterogeneity.
14. Reporting bias assessment	Describe assessment of reporting bias.	Not performed; potential publication bias discussed as mitigated by broad search.
15. Certainty assessment	Describe certainty/confidence assessment.	Not performed; justified for qualitative synthesis with limited studies.
16a. Study selection	Present results of search and selection.	255 identified → 15 full texts → 3 included; Figure 1 (PRISMA diagram).
16b. Excluded studies	List excluded studies and reasons.	Not individually listed; excluded if not case report/series or robotic technique.
17. Study characteristics	Present characteristics of included studies.	Table 2 and narrative description of included reports.
18. Risk of bias in studies	Present bias assessments per study.	Not applicable—case reports; limitation addressed in Discussion.
19. Results of individual studies	Present study-level results.	Narrative summaries of Frey et al., Day et al., Özkan et al.
20. Results of syntheses	Summarize findings across studies.	All demonstrated feasibility and favorable outcomes; discussed qualitatively.
21. Reporting biases	Present assessments of bias due to missing results.	No missing data detected; discussed as limitation of small sample size.
22. Certainty of evidence	Present overall certainty in body of evidence.	Qualitative confidence discussed; evidence rated as limited due to case-level data.
23a. Discussion—interpretation	Interpret results in context of prior evidence.	Discussion contextualizes findings across thoracic and reconstructive surgery.
23b. Discussion—limitations of evidence	Discuss evidence limitations.	Small number of studies, case-level evidence; lack of comparative trials.
23c. Discussion—limitations of review process	Discuss limitations of review methods.	Acknowledged limited evidence and inability to conduct meta-analysis.
23d. Discussion—implications	Discuss implications for practice and research.	Proposes multicenter collaboration and registry development.
24a. Registration and protocol	Provide registration info.	No review protocol registered; PRISMA compliance stated.
24b. Protocol access	State whether a protocol was prepared.	Protocol not prepared; stated in Methods.
24c. Protocol amendments	Describe any protocol amendments.	Not applicable; no amendments made.
25. Support	Describe funding and sponsor roles.	Declarations—‘No external funding was received.’
26. Competing interests	Declare conflicts of interest.	Declarations—‘Authors declare no competing interests.’
27. Data, code, materials	Report data/code availability.	Declarations—‘Data available upon reasonable request.’
